# Modelling the Compaction Step of a Platform Direct Compression Process

**DOI:** 10.3390/pharmaceutics14040695

**Published:** 2022-03-23

**Authors:** Raghu V. G. Peddapatla, Conor Slevin, Gerard Sheridan, Caoimhe Beirne, Shrikant Swaminathan, Ivan Browning, Clare O’Reilly, Zelalem A. Worku, David Egan, Stephen Sheehan, Abina M. Crean

**Affiliations:** 1 SSPC Pharmaceutical Research Centre, School of Pharmacy, University College Cork, T12 K8AF Cork, Ireland; r.peddapatla@umail.ucc.ie (R.V.G.P.); a.crean@ucc.ie (A.M.C.); 2 Alkermes Pharma Ireland Limited, N37 EA09 Athlone, Ireland; conorslevin93@hotmail.com (C.S.); gerrysheridan1990@gmail.com (G.S.); beirneca@tcd.ie (C.B.); ivanbrowning1@gmail.com (I.B.); clare.oreilly@helsinn.com (C.O.); zelalem.worku@alkermes.com (Z.A.W.); 3 Pharmaceutical Manufacturing Technology Centre (PMTC), Bernal Institute, University of Limerick, V94 T9PX Limerick, Ireland; david.egan@ul.ie; 4 Alkermes Inc., Waltham, MA 02451, USA; shrikant_swaminathan@amat.com

**Keywords:** process model, platform process, direct compression, formulation development, porosity, tensile strength, process optimisation, compaction, drug development

## Abstract

The ability to predict formulation behaviour at production scale during formulation design can reduce the time to market and decrease product development costs. However, it is challenging to extrapolate compaction settings for direct compression formulations between tablet press models during scale-up and transfer from R&D to commercial production. The aim of this study was to develop statistical process models to predict tablet tensile strength, porosity and disintegration time from compaction parameters (pre-compression and main compression force, and press speed), for three formulations, with differing deformation characteristics (plastic, brittle and elastic), on three tablet press models (one pilot-scale tablet press (KG RoTab) and two production-scale presses (Fette 1200i and GEA Modul P)). The deformation characteristics of yield pressure and elastic recovery were determined for the model placebo formulations investigated. To facilitate comparison of dwell time settings between tablet press models, the design of experiments (DoE) approach was 9 individual 16-run response surface DoEs (3 formulation × 3 press models), whose results were combined to create a polynomial regression model for each tablet property. These models predicted tablet tensile strength, porosity and disintegration time and enabled the construction of design spaces to produce tablets with specified target properties, for each formulation on each press. The models were successfully validated. This modelling approach provides an understanding of the compaction behaviour of formulations with varying deformation behaviour on development and commercial tablet press models. This understanding can be applied to inform achievable production rates at a commercial scale, during the formulation development.

## 1. Introduction

Pharmaceutical formulation design relates to the development of a drug product that consistently delivers a target product profile with the required quality. Formulation design and process development are interlinked activities; a formulation cannot become a commercial drug product unless it can be produced at a commercial scale. Understanding relationships between formulation and process parameters is a key aspect that underpins process design and control strategies. Process modelling facilitates an understanding of these relationships and enables the identification of the target process parameters that achieve the defined product quality attributes. Process modelling allows the analysis of complex interactions between formulation and process parameters and the generation of process knowledge that can then be applied to assure product quality [1,2,3,4].

Formulation and process design are initially conducted on pilot-scale equipment and then extrapolated to commercial-production-scale equipment. For direct compression formulations, differences between tablet press models/configurations can challenge the extrapolation of process understanding and process models from pilot to production scale [2]. Process models and subsequent design spaces can be scale and equipment dependent. To employ a process model or design space model developed at pilot scale to commercial-scale production, justification is required considering geometric, kinematic and heat and mass transfer differences [5]. When process models are unable to translate between equipment models and production scale, additional experimentation at the production scale is required to augment formulation/process understanding. These additional studies can prolong the product development process, delay time to market and increase development costs. A development approach in which the formulation design process occurs independently, without the consideration of commercial-scale process development, is prone to the risk of an iterative formulation design process. Formulation alterations, during any stage of the clinical development process, that generate pivotal clinical data can result in bridging studies to support formulation safety and bioavailability [6]. Progressing a formulation with consideration of commercial production can result in reduced production output to meet quality requirements [2].

A platform processing approach is reported to accelerate drug product development in the biopharmaceutical sector. A platform approach is the application of common processes to develop a family of similar products [7]. The advantages of employing a platform process for development include a decrease in development time, cost and simplification of both scale-up and process [8]. While the term ‘platform processes’ has not been widely utilised or reported in relation to tablet formulation development, it is common practice during formulation and process development to build on explicit and tacit knowledge achieved from the prior development and production of similar products. In recent years, mechanistic and hybrid modelling approaches have been applied to support oral solid dose development and scale-up [9,10,11]. Such modelling approaches support a platform approach to tablet product development based on explicit rather than tacit knowledge. In this study, we present a statistical process modelling approach to support a platform approach for the compaction stage of the direct compression process. An earlier study by Peddapatla et al. [2] employed a similar statistical modelling approach to predict tablet weight variability for the die filling stage of the direction compression process.

Direct compression tablet production is considered the simplest process for tablet production by the manufacturing classification systems, class 1 [12]. Despite its simplicity, tablet production via the direct compression process is less commonly employed compared to the more complex process of tabletting via a granulation step. Direct compression is more commonly employed for class 1/3 drugs categorised by the biopharmaceutical classification system compared to class 2/4 [13]. The production of tablets by direct compression involves three stages: filling, compaction and ejection. The compaction step influences the tensile strength of the tablets produced. Tensile strength is an important quality attribute that affects the structural integrity of the tablets during coating and packing and other quality attributes such as friability, disintegration and dissolution. Tablet tensile strength depends on the consolidation mechanism of the formulation. The combined effect of process parameters (pre-compression force, main compression force, press speed) and formulation deformation mechanism (plastic, elastic or brittle) determines the tablet tensile strength and other related attributes [14]. A study by Zimmermann et al. looked at the effect of the formulation deformation mechanism (three formulations) on the performance of single punch (four presses) and rotary tablet presses (five presses) from different vendors [15]. The study found that the lowest variation in tablet properties was found for the plastically deforming formulation when compared to an elastically deforming formulation. Moreover, the brittle formulation showed high tablet throughput with acceptable tablet tensile strength. The compression force and rate is a key parameter to maintain for the scale-up of tablet compression process and the transfer of tablet formulation between press models. It is particularly important for shear-rate-sensitive formulations and more pronounced for plastic formulations compared to brittle ones. The dwell time is taken into account when transferring a formulation from a pilot-scale to a production-scale tablet press. During scale-up, tablet press speed is maximised to increase production, while keeping the dwell time within a range that produces tablets with acceptable tensile strength [16]. However, the dwell-time of pilot-scale presses at their fastest production speeds may often equate to the dwell-time of production-scale tablet presses at their lower production speeds, which makes it challenging to maximise the production rate of these products upon scale-up while maintaining tensile strength [2,15,16].

The aim of this study was to elucidate the variation in compaction behaviour of formulations with differing deformation properties on tablet presses with differing of compression force and rate ranges during operation. Firstly, three placebo formulations were designed to mimic the plastic, elastic and brittle deformation behaviour of direct compression formulations. A statistical modelling approach was undertaken to investigate the impact of compaction parameters (pre-compression and main compression force, and press speed) on variation tablet properties (tablet tensile strength, porosity and disintegration time) for three formulations, on three tablet press models: one pilot-scale tablet press (KG RoTab) and two production-scale presses (Fette 1200i and GEA Modul P). To enable the comparison of formulation compaction a single dwell-time between tablet presses, a series of nine individual response surface DoEs were conducted to consider each formulation type and press type combination. Data from these DoE runs were combined to establish polynomial regression models to predict tablet properties of porosity, tensile strength and disintegration time.

## 2. Materials and Methods

### 2.1. Formulation Design

The three placebo formulations used in the development of the process model were designed to exhibit a range of plastic, elastic and brittle deformation behaviour observed in proprietary direct compression formulations. Yield pressure and thickness recovery were determined for each formulation as parameters to classify blend deformation behaviour. The composition of each formulation is described in Table 1. All excipients employed in this study were of commercial excipient grade, meeting the excipient monograph listed in the USP-NF. Formulation 1 (plastic) was designed to show predominantly plastic deformation by including a high proportion of microcrystalline cellulose (87.3 *w*/*w*), which undergoes plastic deformation [14,17,18]. To introduce elastic behaviour in formulation 2 (elastic), an excipient with known elastic behaviour was included, i.e., starch (20% *w*/*w*) [14,18]. A high proportion of lactose (87.3 *w*/*w*), which undergoes brittle deformation, was included in formulation 3 (brittle) to achieve predominantly brittle behaviour [14,17,18].

### 2.2. Formulation Characterisation

Preliminary compaction experiments were conducted to characterise the deformation properties of formulations, using the instrumented laboratory tablet press (Model GTP-1, Gamlen Tableting Ltd., Nottingham, UK). The studies were performed under force control in the fixed-load mode of the tablet press, using a displacement speed of 60 mm/s and a tablet die diameter of 6 mm, in which the compaction force selected by the user is applied using the standard V-shaped compression profile at 1 mms^−1^. The data collection frequency was 200 Hz. The Heckel equation was applied to compaction data to determine the yield pressure for the three formulations to discriminate the plastic and brittle behaviour by comparing yield pressures. Yield pressures were determined by out-of-die compact measurements. The elastic recovery of the tablets was measured by assessing the change in tablet thickness of three formulations immediately after compression and again at the 24 h time point.

### 2.3. Tablet Production

The total weight of each blend prepared was approximately 75 kg. Excipients were dispensed according to the formulation type (Table 1) and passed through a 450 µm sieve (Sweco Europe S.A., Nivelles, Belgium) to remove agglomerates. All excipients, except magnesium stearate, were added into a 100 L IBC (Intermediate Bulk Container, Coleshill, UK), which was attached to the drive of the blender unit (Pharmatech, Coleshill, UK) via a clamping system. The IBC was rotated for 18 min at 20 rpm, for a total of 360 revolutions. Magnesium stearate was then added to the other components in the IBC and blended for an additional 3 min at 20 rpm, for a total of 60 revolutions. Tablets of each formulation blend were produced on one pilot-scale rotary tablet press (KG RoTab (KG-Pharma, Scharbeutz, Germany)) and two production-scale rotary tablet presses (Fette 1200i (Fette Compacting, Schwarzenbek, Germany) and Modul^™^ P (GEA, Bergensesteenweg, Belgium)). Some key technical differences between the tablet presses selected, including tooling details, are listed in Table S1.

Tablet blends were compressed using shield-shape punches to a target tablet weight of 240 mg. The feeder speed on each press was varied to achieved reproducible die fill and, once identified, was maintained constant for each press for all DoE runs. Target average tablet weight was obtained for all formulations on the three tablet presses, and good weight control was observed with %RSD < 2. For the tabletability study, the three placebo formulations were compressed on the three tablet presses, over a compression range of 1.5 to 20 kN (20–360 MPa) at a pre-compression force of 1 kN and at press speed of 11,400 TPH (KG RoTab) and 50,000 TPH (Fette1200i and Modul P). For the individual DoE, the process parameters investigated were pre-compression force, main compression force and press speed, as described in Section 2.4. Approximately 3 kg of blend was compacted for each DoE run.

### 2.4. Design of Experiment

The DoE methodology employed consisted of an individual response surface DoE for each formulation on each tablet press, resulting in 9 (3 × 3) DoEs. The DoE design was carried out using JMP statistical software (Version 13, SAS Institute, Inc., Cary, NC, USA). The objective of the individual DoE was to identify combinations of pre-compression force, main compression force and press speed for each formulation on each tablet press design, at which tablets with specified quality attributes could be produced. This DoE approach was undertaken in preference to a single DoE with 5 factors (pre-compression force, main compression force, press speed, press type and formulation type). The DoE approach used enabled the comparison of dwell times between tablet press types and facilitated the inclusion of data related to additional formulation and press types in a structured way at a later time point. The target tablet properties selected for this study were tensile strength, 1 to 3 MPa; porosity 7% to 15% and disintegration time <5 min (<300 s). The tensile strength values are in agreement with the general guide for finished tablets of >1.7 MPa at a solid fraction of 0.9 proposed by the manufacturing classification system [12].

Once the required operating ranges were identified, maximum production rates for each formulation on each press could be determined. The specified tablet quality attributes included tablet weight, thickness, hardness, porosity, tensile strength and disintegration time and the quality attributes modelled included tablet tensile strength, porosity and disintegration time. A 16-run response surface design, including 2 centre points was performed for each formulation, on each tablet press. The cube plot and DoE run order of experiments is shown in the supplementary information (Figure S1 and Table S2).

The DoE parameters and levels are detailed in Table 2. Three levels of pre-compression force, 1, 2.5 and 5 kN, were investigated for all formulations on all tablet presses. The compression force levels investigated in DoE were determined from tabletability studies conducted for each formulation on each press (Section 2.3). Compression forces that produced tablets with porosity values of approximately 10%, 15% and 20% were selected as the low, medium and high compression force levels (Table S3). The tablet press speed levels investigated also varied across the tablet press models and were based on their respective minimum and maximum press speeds. For each tablet press, at least one press speed level was set to match another press based on their dwell times, as shown in Table 2.

### 2.5. Measurement of Tablet Properties

Twenty tablets were analysed using a Smart-test 50 Autotester (Pharmatron, Aesch, Switzerland). Mean tablet weight, thickness, hardness and variability (%RSD) were measured for every 20 tablets collected per 1 kg blend compacted. The disintegration time was measured for tablets collected after approximately 1.5 kg of blend being compacted. Tablet disintegration was evaluated with a USP disintegration tester (DISI-M tester, Zuchwil, Switzerland) using distilled water at 37 °C. Tablet porosity was measured using Equation (1) and the tensile strength of the tablets was calculated using Equation (2) [19].

(1) $$\left(1-Tablet\;Solid\;Fraction\right)\times 100$$

(2) $$\sigma_{t}=\frac{2\;}{3}\;\left(\frac{10P}{\pi D^{2}\left(2.84\frac{t}{D}-0.126\frac{t}{W}+3.15\frac{W}{D}+0.01\right)}\right)$$

where *σ_t_* is tensile strength, *P* is hardness, *D* is the length of the short axis, *t* is the overall thickness and *W* is the wall height of the tablet.

### 2.6. Data Analysis

Data from the 144 DoE runs (9 DoEs × 16 runs) were combined to establish polynomial regression models to predict the tablet properties of porosity, tensile strength and disintegration time. Data were analysed, and outliers were manually identified and removed: 2 outliers removed from the tensile strength and porosity data and 3 outliers from the disintegration time data. To enable the DoE results to be combined into a single polynomial model for each tablet property, press speeds and compression force settings for different presses (Table 2) were standardised. The standardised parameters were referred to as the ‘Coded’ press speed and the main compression force, respectively. Standardisation was performed by subtracting the mean value of the parameter across DoE runs from the actual run value and dividing by the standard deviation. Model development was carried out using JMP statistical software (Version 13, SAS Institute, Inc., Cary, NC, USA). Input process data (tablet press model, formulation type, pre-compression force, coded main compression force and coded press speed) and tablet properties (tablet tensile strength, porosity and disintegration time) were imported into JMP. All continuous factors were centred during the analysis. Polynomial regression models considering linear, 2-way, 3-way, 4-way interactions and quadratic terms were created using fit model analysis in JMP. The models identified statistically significant input factors effecting tablet tensile strength, porosity and disintegration time.

## 3. Results

### 3.1. Deformation Behaviour of Placebo Blends

A key element of the experimental design was the inclusion of formulations with a range of deformation characteristics and, hence, the requirement to design placebo formulations that mimic the deformation behaviour of direct compression formulations [14,20,21,22]. The yield pressure (P_y_) and elastic recovery of formulations were determined to confirm differences in compaction behaviour. The Heckel analysis was used to differentiate plastic deformation from brittle fracture. Materials that undergo plastic deformation show a relatively higher slope with lower yield pressure compared to those that undergo brittle fracture [21]. The yield pressures for the three different formulations determined from compaction studies on the Gamlen and KG RoTab are shown in Table 1. The yield pressure of formulation 1 was lower than that of formulations 2 and 3, indicating increased plasticity, which can be attributed to the higher concentration of MCC [14]. A high concentration of lactose was included in formulation 3, which is a brittle material with a fragmentation tendency [14,18]. Lactose is reported to exhibit a higher yield pressure compared to MCC. All formulations showed an increase in yield pressure when compacted at relatively higher compaction speeds on the KG RoTab compared to the Gamlen bench-top press. These differences were attributed to formulation strain rate sensitivity. The greatest increase in yield pressure was noted for formulation 2, which contained a diluent of 20% starch in a lactose/MCC mix. Starch was added as an elastic component, and this increase in yield pressure at faster tableting speeds can also be caused by changes in tablet porosity upon ejection, which has been observed during out-of-die compression analysis.

To determine the formulation’s elastic recovery, tablets of each formulation compressed to 20% porosity on the Modul P tablet press were selected. Thickness readings were recorded immediately after compaction and again after 24 h. Among the three formulations, formulation 2 showed significant elastic recovery after 24 h, indicating elastic behaviour due to the presence of starch within the formulation 2 (Table 1).

**Figure 1:**
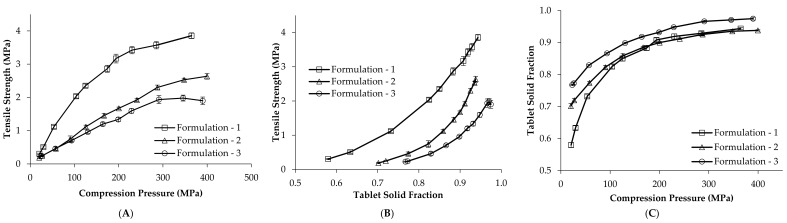
(**A**) Tabletability profile, (**B**) compactibility profile and (**C**) compressibility profile of three placebo formulations (formulation 1 (plastic), formulation 2 (elastic) and formulation 3 (brittle) on the KG RoTab tablet press at a press speed of 11,400 TPH. Average values are shown, *n* = 10, the y error bar indicates standard deviation.

To further characterise the compaction properties of the formulations, the tabletability, compactibility and compressibility profiles were constructed for each formulation on the KG RoTab, at a press speed of 11,400 TPH, and are shown as an example, Figure 1. Tabletability describes the cause–effect relationship between the compression pressure and tablet strength [14,23,24]. Tabletability is dependent on the formulation’s deformation properties and the applied compression pressure and rate of application, which can vary with both press speed and press model. At similar compression forces, formulation 1 (plastic) showed the highest tensile strength, Figure 1A. The compactibility profiles show the intrinsic relationship between tablet tensile strength and solid fraction for individual formulations, which is independent of the applied compression used to produce tablets [24]. Among the three formulations, formulation 1 (plastic) showed the highest compactibility and formulation 3 (brittle) showed a slightly lower compactibility compared to formulation 2 (elastic), Figure 1B. Compressibility profiles assess the change in volume of the powder blend when it is compressed [23,24]. Compressibility profiles were used to inform the high, medium and low main compression force levels investigated in the individual DoEs (Section 2.4). Compression pressures (and related compression forces) that produced tablets with solid fractions of approximately 0.9, 0.85 and 0.8 (porosity 10%, 15% and 20%) for each formulation on each press were selected, Table 2.

**Figure 2:**
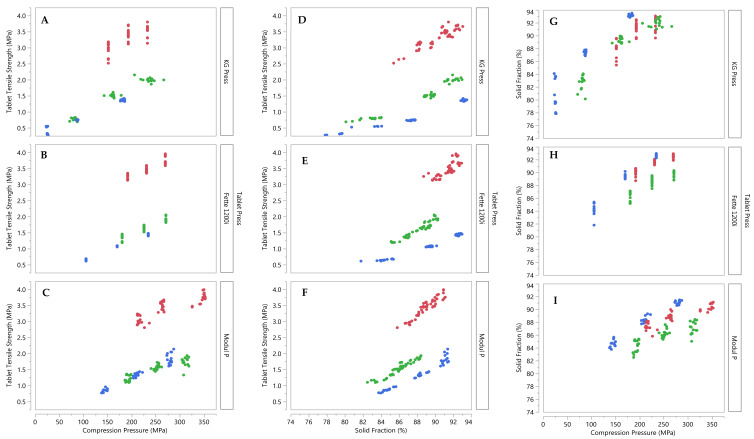
Compaction behaviour of three placebo formulations compressed on three tablet presses using processing parameters defined by the DoE. Red circles indicate formulation 1 (plastic), green circles indicate formulation 2 (elastic) and blue circles indicate formulation 3 (brittle). (**A**–**C**) Tabletability profiles, (**D**–**F**) compactibility profiles and (**G**–**I**) compressibility profiles of the three formulations on three tablet presses.

The tabletability, compactibility and compressibility profiles of the formulations were also analysed from the compaction data of the individual DoE runs. Compared to the profiles shown in Figure 1, these profiles show the formulation’s behaviour across a range of pre-compression forces and press speeds. The tabletability profiles again show greatest tensile strength for formulation 1 (plastic) at similar compression pressures, Figure 2A–C. More variability was observed for formulation 1 compared to that for other formulations, particularly on the KG Press. Figure 2D–F depicts the compactibility profiles, which showed similar compactibility behaviour to that in Figure 1B on all three tablet presses. Figure 2G–I shows the compressibility profiles. On the KG press, formulation 3 (brittle) resulted in tablets with the highest solid fractions at equivalent compression pressures, and formulation 1 (plastic) and formulation 2 (elastic) showed similar compressibility profiles equivalent compression pressures. The greatest variability in tablet solid fraction was observed for formulation 1 (plastic) on the KG press. These differences cannot be attributed solely to the reduced press speeds and higher dwell times for the KG press (33–66 ms). DoE were designed for overlap in dwell-times across press models; dwell-times investigated on the Fette 1200i were 7–33 ms and on the Modul P they were 7–21 ms, respectively.

### 3.2. Development of Tablet Tensile Strength, Porosity and Disintegration Time Models

Models were developed to predict the process parameters for each press that would produce tablets with specified target properties (tensile strength, porosity and disintegration time). Due to differences in achievable press speeds, compaction mechanisms and, therefore, dwell times across press models, different main compression forces are required on different presses to achieve equivalent tablet tensile strength and porosity. To develop process models for each tablet property that can support a platform development of formulations, the nine individual DoEs (three formulations on tree presses), investigating three factors (pre-compression, main compression and press speed), were combined into single models. To enable the inclusion of the different press speeds and compression forces investigated across the individual DoEs (Table 2), the actual values were standardised and coded as described in Section 2.6. The combined process model identified all factors, their interactions (two-, three-, four- and five-way interactions) and quadratic terms that had a significant effect on tablet tensile strength, porosity and disintegration time.

Fit model analysis predicted the best fit for the log transformation for tensile strength and porosity and square root transformation for disintegration time. The regression models obtained were statistically significant, and non-significant factors were removed from the fit model. A summary of the statistical parameters for the reduced regression model is shown in Table 3. Effects analyses for the transformed tablet tensile strength, porosity and disintegration time model are shown in Table 4. Individual factors were significant in all models, with the exception of pre-compression force in the disintegration model. Coded press speed had a significant quadratic effect in the tensile strength model, and pre-compression force had a significant quadratic effect for both the tensile strength and porosity models. Significant two-, three- and four-way interactions varied between models, and a five-way interaction was significant for the disintegration model.

### 3.3. Design Space

**Figure 3:**
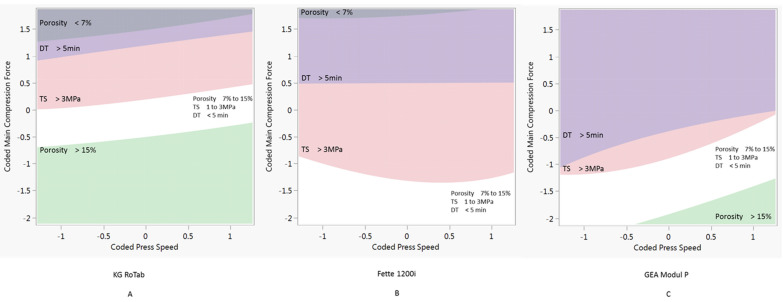
Two-dimensional contour profiles showing optimum process conditions for tablet tensile strength, porosity and disintegration time at optimum coded main compression force and coded press speed for formulation 1 on three tablet presses at a constant pre-compression force of 1 kN. (**A**) KG RoTab, (**B**) Fette 1200i and (**C**) Modul P. Coded main compression force and coded press speed values correspond to standardised units (Section 2.6). DT indicates disintegration time, and TS represents tensile strength. Regions of main compression force and press speed settings at which the tablets produced meet the target specifications of porosity 7% to 15%, tensile strength 1 to 3 MPa and disintegration time <15 min are in white. Regions of main compression force and press speed settings at which the tablets produced were outside the target specification are indicated by different shaded areas.

**Figure 4:**
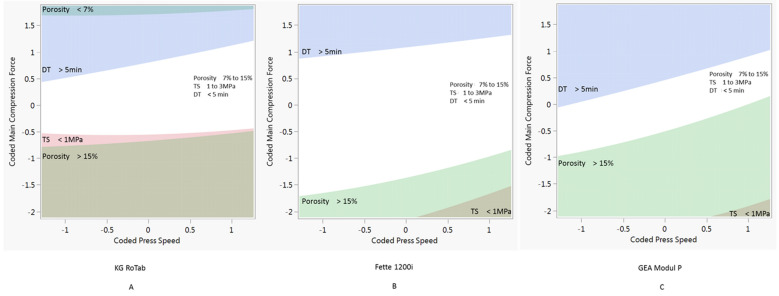
Two-dimensional contour profiles showing optimum process conditions for tablet tensile strength, porosity and disintegration time at optimum main compression force and press speed for formulation 2 on three tablet presses at a constant pre-compression force of 1 kN. (**A**) KG RoTab, (**B**) Fette 1200i and (**C**) Modul P. Coded main compression force and coded press speed values correspond to standardised units (Section 2.6). DT indicates disintegration time, and TS represents tensile strength. Regions of main compression force and press speed settings at which the tablets produced meet the target specifications of porosity 7% to 15%, tensile strength 1 to 3 MPa and disintegration time <15 min are in white. Regions of main compression force and press speed settings at which the tablets produced were outside the target specification are indicated by different shaded areas.

**Figure 5:**
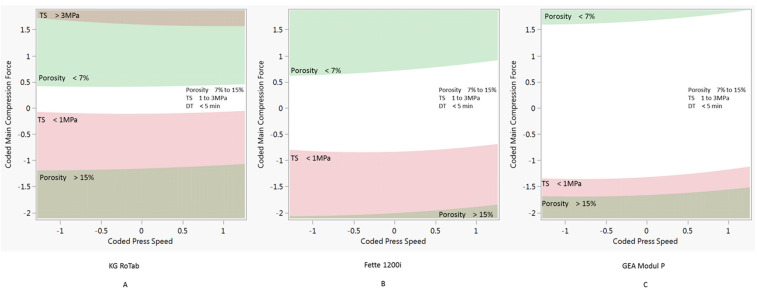
Two-dimensional contour profiles showing optimum process conditions for tablet tensile strength, porosity and disintegration time for formulation 3 at a set pre-compression force of 1 kN. (**A**) KG RoTab, (**B**) Fette 1200i and (**C**) Modul P. Coded main compression force and coded press speed values correspond to standardised units (Section 2.6). DT indicates disintegration time, and TS represents tensile strength. Regions of main compression force and press speed settings at which the tablets produced meet the target specifications of porosity 7% to 15%, tensile strength 1 to 3 MPa and disintegration time < 15 min are in white. Regions of main compression force and press speed settings at which the tablets produced were outside target specification are indicated by different shaded areas.

The process models developed were used to predict process conditions to produce tablets with the target tensile strength, porosity and disintegration time across a range of press speeds. The target tablet properties selected for this study were tensile strength 1 to 3 MPa, porosity 7% to 15% and disintegration time <5 min (<300 s). In this study, the required process conditions were determined keeping the pre-compression force constant at 1 kN. The combinations of process parameters predicted to produce tablets with the target properties are shown as white regions in Figure 3, Figure 4 and Figure 5.

Figure 3 shows the design space for formulation 1 (plastic) on the three presses. Due to the tabletability of the formulation 1 (Figure 2), there is a relatively narrow window of compression force at all three press speeds. The design space for formulation 2 (elastic) is shown in Figure 4. Compared to the plastic formulation 1, the elastic formulation 2 had a wider compression window on the KG Rotab and Fette 1200i. As press speed increased, the required compression force increased for both production-scale presses but not for the pilot-scale KG RoTab. This can be attributed to the lower-range press speeds and dwell times investigated on KG RoTab. Figure 5 shows the optimum conditions for formulation 3 (brittle). The window of optimum compression force was the greatest in Modul P, followed by that in the Fette 1200i, with a narrow window on the KG RoTab. Compared to formulation 2 (elastic), the required compression force for formulation 3 (brittle) was not highly dependent on tablet press speed for the three presses.

The properties constraining the predicted processing conditions varied depending on formulation’s deformation type. For example, higher compression forces resulted in tablets of formulation 1 (plastic) exceeding the target tensile strength values on all presses. Lower compression forces resulted in tablets with porosity values above target values on the KG RoTab press and at higher press speeds on the Module P. Formulation 2 (elastic) was constrained by above-target disintegration time at upper compression forces and below-target tensile strength at lower compression forces. Below-target tensile strength and porosity were the constraints for formulation 3 (brittle) at lower and higher compression forces, respectively.

### 3.4. Tensile Strength and Porosity Model Validation

**Figure 6:**
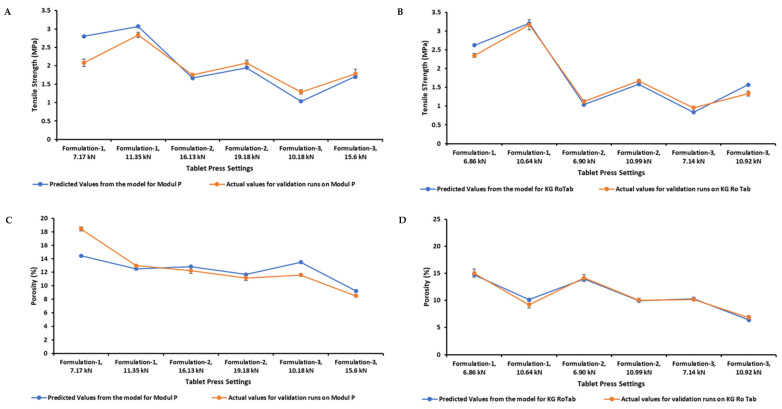
Validation of formulations 1–3 on a production-scale tablet press (Modul P) (**A**,**C**) and a pilot-scale one (KG RoTab) (**B**,**D**). The models were validated at a press speed of 14,400 TPH on KG RoTab and 50,000 TPH on Modul P at two different compression forces for each formulation. Actual values of tensile strength and porosity from the runs for the formulations on two presses were taken and compared to the values predicted using model at similar settings. For actual values, average values are shown, *n* = 10 and the y error bar indicates standard deviation.

The tensile strength and porosity models were validated using mid-range process operating conditions on the KG RoTab and Modul P presses. Formulations 1–3, which were used to develop the models, were also used for model validation. Validation process parameters are listed in the supplementary information (Table S4). The predicted and actual values for porosity and tensile strength for the formulations on two presses are shown in Figure 6. For the three formulations, the process model accurately predicted the porosity values on KG RoTab (Figure 6D). The tensile strength was also accurately predicted, except for a slight overprediction for formulation 1 (plastic) and formulation 3 (brittle) at the higher and lower compression forces, respectively (Figure 6B). Overall, the models’ prediction capacity was less accurate for Module P. The greatest deviation from the predicted values of tensile strength and porosity was observed for formulation 1 (plastic) at the compression force of 7 kN on Modul P (Figure 6A,C).

## 4. Discussion

The objective of this study was to develop a modelling approach to predict how formulations with different deformation mechanisms would behave with press designs and control strategies employed routinely in an industrial setting. This study builds on an earlier study focused on predicting tablet weight variability based on the flow properties of the direct compressible formulations on different tablet presses [2]. In the current study, process models to predict tablet tensile strength, porosity and disintegration time for direct compression formulations on pilot and product press models were developed, to support a platform approach to formulation development. The models were developed with data from placebo formulations designed to exhibit varying deformation characteristics. Blend deformation behaviour was classified categorically based on the yield pressure (Yp) and elastic recovery values [25]. It is noteworthy that the placebo formulations in this study were designed to reflect the relatively minor variation in behaviour that can be encountered in commercial tablet formulations, rather than the higher variation in deformation behaviour previously studied in compaction studies of excipients and binary blends [15,26].

The modelling approach used advances the traditional QbD approach for direct compression, where process-related models and design spaces can be scale and sometimes equipment dependent [5]. It provides a framework to predict tablet quality attributes across different tablet press models of varying scale for formulations with varying deformation characteristics. The modelling approach combines the results of individual DoEs for each press/formulation combination has the capacity to incorporate additional DoEs for extra press models and or formulation types. The models developed are empirical and differ from other advancements in QbD approaches to scale-up which employ mechanistic and hybrid modelling approaches.

The approach undertaken incorporated the tablet press model and the formulation deformation mechanism as categorical variables in the experimental design, and pre-compression force, main compression force and tablet press speed as numerical process parameter variables. The process models developed included linear, quadratic and interaction effects of input parameters on tablet porosity, tensile strength and disintegration time, with $R_{adj}^{2}$ of 99.01%, 97.06% and 95.54%, respectively. The optimum process conditions predicted by the models provide an overview of the behaviour of direct compression formulations with varying deformation behaviours across a range of press types. The tensile strength and porosity models were validated on the KG RoTab and Modul P tablet presses and, overall, provided a good indication of formulation compaction behaviour.

By enabling a company to predict tablet porosity, tensile strength and disintegration time for a range of formulations with differing deformation behaviours across tablet presses in their inventory, the modelling approach undertaken can support formulation development, scale-up and transfer between tablet press models. A platform, direct compression formulation development approach requires process models that adapt process parameters to compensate for changes in formulation deformation behaviour and variation in tablet press operational designs. During development at the pilot scale, the model can be applied to predict optimum production speeds and compaction forces upon scale-up, based on blend deformation properties. This can inform formulation design to maximise productivity without the requirement for extensive production-scale runs. Formulation design during R&D can adjust deformation behaviour with a view to increasing commercial production speeds. During commercial production, understanding differences in formulation deformation behaviour between production-scale presses facilitates the selection of optimum pre-compression force, main compression force and press speed when transferring between presses. The model approach employed can also be used to select a tablet press from an available inventory to maximise tablet production whilst maintaining product quality. The combination of the modelling approaches applied to die fill in our earlier study [2] and the compaction step in this study can support a comprehensive platform formulation development approach that can potentially reduce development costs and time to market.

## 5. Conclusions

This paper presents a modelling approach to support the transfer of direction compression formulations with varying deformation behaviours between pilot- and production-scale tablet presses. The modelling approach involved the development of statistical process models to predict tablet porosity, tensile strength and disintegration time for direct compression formulations. Models were developed using a DoE approach, with pre-compression force, main compression force and press speed as numerical variables and press type and formulation type as categorical variables. Placebo formulations with variation in deformation behaviour (plastic, elastic and brittle) were developed, and the compaction behaviour was characterised. Models were used to establish a main compression force/press speed design space for each formulation, on each press to achieve the target tablet tensile strength, porosity and disintegration time. The modelling approach undertaken can be applied to aid platform formulation development, scale-up and transfer of formulations between production presses.

## Figures and Tables

**Table 1:** Composition, yield pressure and elastic recovery of placebo blends used to develop the process models.

Placebo Blend Composition (% *w*/*w*)				
Components	Function	Formulation 1 Plastic	Formulation 2 Elastic	Formulation 3 Brittle
Microcrystalline cellulose	Diluent	87.3	38.5	9.7
Lactose	Diluent	9.7	38.5	87.3
Starch	Elastic component	0	20	0
Crospovidone	Disintegrant	1	1	1
Colloidal silicon dioxide	Flow aid	1	1	1
Magnesium stearate	Lubricant	1	1	1
**Placebo Blend Characterisation**				
Yield pressure (Py) (MPa)—Gamlen		40	49	76
Yield pressure (Py) (MPa)—KG RoTab		74	141	130
Elastic recovery after compaction (mm)		3.80 ± 0.01	3.98 ± 0.01	3.98 ± 0.01
Elastic recovery after 24 h (mm)		3.81 ± 0.01	4.01 ± 0.01	3.98 ± 0.01

**Table 2:** DoE parameter levels investigated for each formulation on each tablet press: pre-compression force, main compression force, tablet press speed levels and associated dwell times for each tablet press. Form. 1, 2 and 3 refer to formulations 1, 2 and 3.

Tablet Press	Level	Pre-compression Force (kN)	Compression Force (kN)	Tablet Press Speed (Tablets per Hour)	Dwell Time (ms)
KG RoTab	−1	1	8.83 (Form. 1)	9600	66
			4.81 (Form. 2)		
			1.50 (Form. 3)		
	0	2.5	11.16 (Form. 1)	14,400	44
			9.40 (Form. 2)		
			5.14 (Form. 3)		
	1	5	13.50 (Form. 1)	19,200	33
			13.98 (Form. 2)		
			10.56 (Form. 3)		
Fette 1200i	−1	1	11.12 (Form. 1)	28,000	33
			10.45 (Form. 2)		
			6.12 (Form. 3)		
	0	2.5	13.39 (Form. 1)	86,000	11
			13.08 (Form. 2)		
			9.86 (Form. 3)		
	1	5	15.65 (Form. 1)	130,000	7
			15.71 (Form. 2)		
			13.60 (Form. 3)		
Modul P	−1	1	12.40 (Form. 1)	46,000	21
			11.41 (Form. 2)		
			8.29 (Form. 3)		
	0	2.5	15.19 (Form. 1)	93,000	11
			14.27 (Form. 2)		
			12.20 (Form. 3)		
	1	5	17.97 (Form. 1)	140,000	7
			18.03 (Form. 2)		
			16.10 (Form. 3)		

**Table 3:** Summary of the reduced regression models’ statistical parameters.

Statistical Parameter	Model		
	Tensile Strength	Porosity	Disintegration Time
R^2^	0.9918	0.9756	0.9628
Adjusted R^2^	0.9901	0.9706	0.9554
Root mean square error	0.0603	0.0461	1.0124
Observations (or sum weights)	430	430	429

**Table 4:** Effect summary of model terms (factors) and their interactions showing significant effects (*p* < 0.05) on the log-transformed tensile strength, porosity and square-root-transformed disintegration time. NS indicates not significant (*p* > 0.05).

Model Terms	Tensile Strength Model	Porosity Model	Disintegration Time Model
Tablet press	<0.0001	<0.0001	<0.0001
Formulation	<0.0001	<0.0001	<0.0001
Coded main compression force	<0.0001	<0.0001	<0.0001
Coded press speed	<0.0001	<0.0001	<0.0001
Pre-compression force	<0.0001	<0.0001	NS
Coded press speed * Coded press speed	<0.0001	NS	NS
Pre-compression force * Pre-compression force	<0.0001	<0.0001	NS
Tablet press * Formulation	<0.0001	<0.0001	<0.0001
Tablet press * Coded main compression force	<0.0001	<0.0001	<0.0001
Tablet press * Coded press speed	<0.0001	NS	<0.0001
Tablet press * Pre-compression force	<0.0001	0.0023	NS
Formulation * Main compression force	<0.0001	<0.0001	<0.0001
Formulation * Coded press speed	NS	<0.0001	<0.0001
Formulation * Pre-compression force	NS	0.014	NS
Coded main compression force * Coded press speed	0.0006	NS	NS
Coded main compression force * Pre-compression force	0.0008	0.0005	NS
Coded press speed * Pre-compression force	0.0465	NS	<0.0001
Tablet press * Formulation * Coded main compression force	<0.0001	NS	<0.0001
Tablet press * Formulation * Coded press speed	0.0012	NS	NS
Tablet press * Formulation * Pre-compression force	NS	0.0457	NS
Tablet press * Coded main compression force * Coded press speed	NS	NS	0.0023
Tablet press * Coded press speed * Pre-compression force	0.0117	NS	NS
Formulation * Coded main compression force *Coded press speed	NS	NS	0.0091
Formulation * Coded main compression force * Pre-compression force	<0.0001	0.0056	NS
Tablet press * Formulation * Coded main compression force * Coded press speed	NS	NS	0.0058
Tablet press * Formulation * Coded main compression force * Pre-compression force	<0.0001	0.0029	<0.0001
Tablet press * Coded main compression force * Coded press speed pre-compression force	0.0265	NS	0.0014
Formulation * Pre-compression force * Coded main compression force * Coded press speed	0.0349	NS	NS
Tablet press * Formulation * Pre-compression force (kN) * Main compression force * Press speed	NS	NS	0.0323

* Indicates interaction between the factors.

## Data Availability

Data available on request due to restrictions.

## Supplementary Materials

The following supporting information can be downloaded at: https://www.mdpi.com/article/10.3390/pharmaceutics14040695/s1: Table S1. Key technical specification of tablet press in this study with specific type of tooling. Table S2. DoE run order of experiments for three different formulations on three tablet presses. Table S3: Main compression force applied to achieve the target tablet porosities of 10%, 15% and 20% for each formulation on each tablet press. Table S4. Process parameters used to validate the models. Figure S1. Response surface design DoE with 14 runs (green) and 2 centre points (blue).
