# Supplementary material for: Modelling the Compaction Step of a Platform Direct Compression Process
Source: Pharmaceutics. 2022 Mar 23;14(4):695. doi: 10.3390/pharmaceutics14040695 (PMC9027228; doi:10.3390/pharmaceutics14040695)
Supplement: Supplementary file 1 [file pharmaceutics-14-00695-s001.zip › pharmaceutics-1602834-supplementary.pdf]

pharmaceutics

Supplementary Materials: Modelling the Compaction Step of a Platform Direct Compression Process

Raghu V. G. Peddapatla, Conor Slevin, Gerard Sheridan, Caoimhe Beirne, Shrikant Swaminathan, Ivan Browning, Clare O'Reilly, Zelalem A. Worku, David Egan, Stephen Sheehan and Abina M. Crean

Table S1. Key technical specification of tablet press in this study with specific type of tooling.

Parameters	KG RoTab	Fette 1200i	GEA Modul P
No of punch stations	8	24	21
Max tablet output per hour (TPH)	19,200	140,000	140,000
Fill weight control mechanism	Main compression force measurement	Main compression force measurement	Pre-compression displacement at equal force
Feed frame design (no. chambers)	2 chambers	3 chambers	2 chambers
Punch type	D	B	B
Punch Shape	Shield (Triangular)		
Tip edge width (mm)	8.5		
Perimeter of punch tip (mm)	27.8		
Cross sectional area of punch tip (mm^2)	54.8		

Table S2. DoE run order of experiments for three different formulations on three tablet presses. The centre points are shown in bold.

Runs	Pre-Compression Force	Main Compression Force	Press Speed
1	−1	−1	+1
2	1	1	+1
3	−1	1	−1
4	−1	1	+1
5	1	−1	+1
6	0	−1	0
7	−1	−1	−1
**8**	**0**	**0**	**0**
9	−1	0	0
**10**	**0**	**0**	**0**
11	0	0	+1
12	1	1	−1
13	0	1	0
14	1	−1	−1
15	1	0	0
16	0	0	−1

Table S3. Main compression force applied to achieve the target tablet porosities of 10%, 15% and 20% for each formulation on each tablet press.

Tablet Press	Formulation	Target Porosity (%)	Main Compression Force (kN)
KG RoTab	1	10	13.50
		15	11.16
		20	8.83
	2	10	13.98
		15	9.40
		20	4.81
	3	10	10.56
		15	5.14
		20	1.50
Fette 1200i	1	10	15.65
		15	13.39
		20	11.12
	2	10	15.71
		15	13.08
		20	10.45
	3	10	13.60
		15	9.86
		20	6.12
GEA Modul P	1	10	17.97
		15	15.19
		20	12.40
	2	10	18.03
		15	14.27
		20	11.41
	3	10	16.10
		15	12.20
		20	8.29

Table S4. Process parameters used to validate the models.

Tablet Press	Formulation	Main Compression Force (kN)	Pre compression Force (kN)	Tablet Press Speed (TPH)
KG RoTab	Plastic	6.86	1	14,400
	Plastic	10.64	1	14,400
	Elastic	6.9	1	14,400
	Elastic	10.99	1	14,400
	Brittle	7.14	1	14,400
	Brittle	10.92	1	14,400
GEA Modul P	Plastic	7.17	1	50,000
	Plastic	11.35	1	50,000
	Elastic	16.13	1	50,000
	Elastic	19.18	1	50,000
	Brittle	10.18	1	50,000
	Brittle	15.60	1	50,000

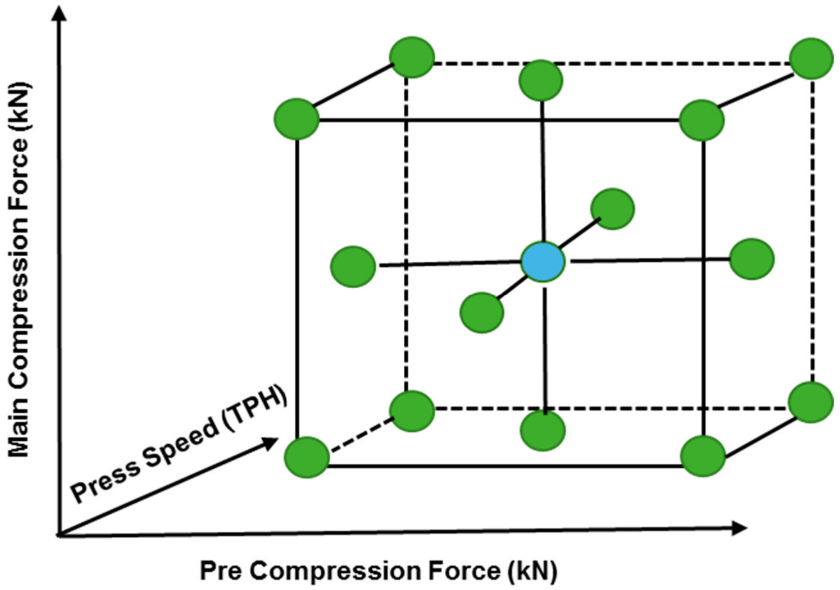

Figure S1. Full response surface design DoE with 14 runs (green) and 2 centre points (blue).
